# Cancerous pericarditis presenting as cardiac tamponade in a 68-year-old man with pancreatic adenocarcinoma: a case report

**DOI:** 10.1186/s13256-020-02546-y

**Published:** 2020-11-07

**Authors:** Sachie Kiryu, Zensho Ito, Masashi Ishikawa, Takafumi Akasu, Yoshihiro Matsumoto, Shinichi Hirooka, Masayuki Saruta, Shigeo Koido

**Affiliations:** 1 Division of Gastroenterology and Hepatology, Department of Internal Medicine, The Jikei University School of Medicine, Kashiwa Hospital, 163-1 Kashiwa-shita, Kashiwa, Chiba 277-8567 Japan; 2 Department of Pathology, The Jikei University School of Medicine, Kashiwa Hospital, Kashiwa City, Chiba 277-8567 Japan; 3 Division of Gastroenterology and Hepatology, Department of Internal Medicine, The Jikei University School of Medicine, Tokyo, 105-8461 Japan

**Keywords:** Pancreatic cancer, Cardiac tamponade

## Abstract

**Introduction:**

Pericardial effusion is a rare complication of pancreatic cancer. We report a case of cardiac tamponade secondary to pancreatic cancer.

**Case presentation:**

A 68-year-old Japanese man was diagnosed as having pancreatic cancer during surgery and received chemotherapy for 28 months after the diagnosis. He was admitted to the emergency room with severe dyspnea. Echocardiography revealed pericardial effusion with severe hypofunction. Emergency pericardial drainage was performed to maintain hemodynamics, which resulted in the elimination of 450 mL of blood and the maintenance of circulatory dynamics. Cytological examination of the pericardial fluid revealed atypical cells and tumor cells suggesting adenocarcinoma.

**Conclusions:**

To our knowledge, pancreatic cancer complicated with cancerous pericarditis has not been previously documented. This case highlights the extreme severity of pericardial effusion, a sign of progressive disease, secondary to pancreatic cancer. In the case of neoplastic pericardial effusion, an extremely poor prognosis must be considered.

## Introduction

Malignant pericardial effusion caused by carcinomatous pericarditis is a complication of advanced malignancy. Pericardial effusion may cause cardiac tamponade and sudden death if not properly controlled. Cardiac tamponade as a complication of pancreatic cancer is very rare. Cardiac tamponade caused by carcinomatous pericarditis induces the retention of pericardial fluid, causing pericardial effusion, and must be treated promptly as an oncologic emergency [1]. Emergency treatment is necessary to prevent sudden death and relieve symptoms of pericardial effusion in patients with neoplastic cardiac tamponade [2]. To the best of our knowledge, pericardial involvement in patients with pancreatic cancer has never been reported. Here, we report a case of metastatic pancreatic cancer complicated by pericardial effusion.

## Case report

**Fig. 1:**
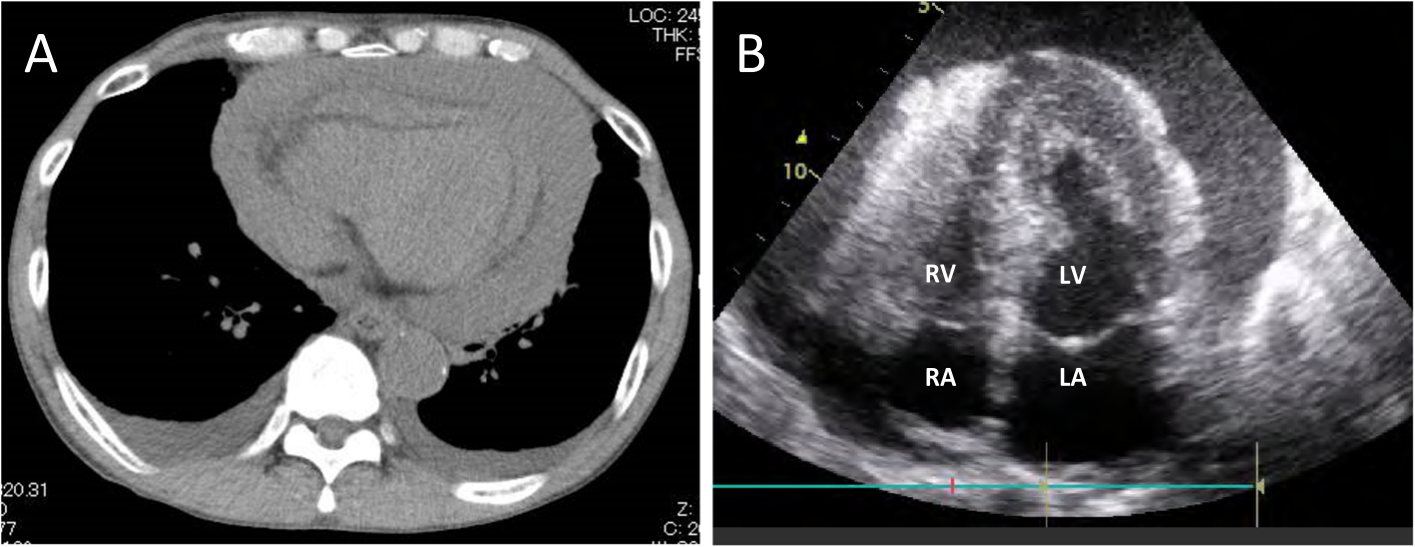
**a** Computed tomography findings. Extensive pericardial effusion and bilateral pleural effusion. **b** Echocardiography (apical four-chamber view) findings. An echo-free space around the heart revealed severe pericardial effusion. *LA* left atrium, *LV* left ventricle, *RA* right atrium, *RV* right ventricle

A 68-year-old Japanese man was suspected of having pancreatic cancer 3 years previously due to an increase in carbohydrate antigen (CA)19-9. Abdominal computed tomography (CT) revealed a 26-mm hypoechoic mass on the head of his pancreas and liver metastasis. Endoscopic ultrasonography (EUS) and fine-needle aspiration (FNA) of the pancreatic mass revealed adenocarcinoma. He was diagnosed as having Stage IV unresectable pancreatic cancer, and gemcitabine (GEM) plus nanoparticle albumin-bound paclitaxel (nab-PTX) doublet chemotherapy was administered. After 2 years of GEM/nab-PTX chemotherapy, progression of the disease was confirmed; therefore, the chemotherapy regimen was changed to GEM + tegafur-gimeracil-oteracil (S1). Six months later, he experienced dyspnea and visited the clinic after a few days of experiencing symptoms. CT revealed pericardial effusion and bilateral pleural effusion. Laboratory tests revealed elevated C-reactive protein (CRP) at 6.23 mg/dL. Pneumonia was treated with beta-lactam antibiotics. Therefore, he was diagnosed as having pneumonia and heart failure. The following day, his blood oxygen saturation was 90% on room air, and he had tachycardia (110 beats per minute) and hyperthermia (37.2 °C). His blood pressure was 86/67 mmHg. Respiratory and circulatory failure was managed in an emergency room. Electrocardiography revealed sinus tachycardia, and echocardiography revealed a severe pericardial swinging heart motion (Fig. 1a and b). Pericardial drainage was performed through a pericardial window, resulting in the drainage of 450 mL of blood, and the pericardial fluid was subjected to cytological examination. Cytological examination of the fluid revealed tumor cells indicating adenocarcinoma. Blood levels of CA19-9 and carcinoembryonic antigen (CEA) were 5.8 ng/mL and 50 IU/mL, respectively, which gradually increased during the course of treatment, suggesting pancreatic cancer progression. A pericardial drainage tube was removed after improvement in his general condition. One week after the surgical procedure, intravenously administered chemotherapy with GEM/nab-PTX was re-initiated. Sixteen days after re-initiation of chemotherapy, he was admitted to the emergency room and died of cardiac arrest due to severe respiratory failure without evidence of recurrent pericardial effusion.

**Fig. 2:**
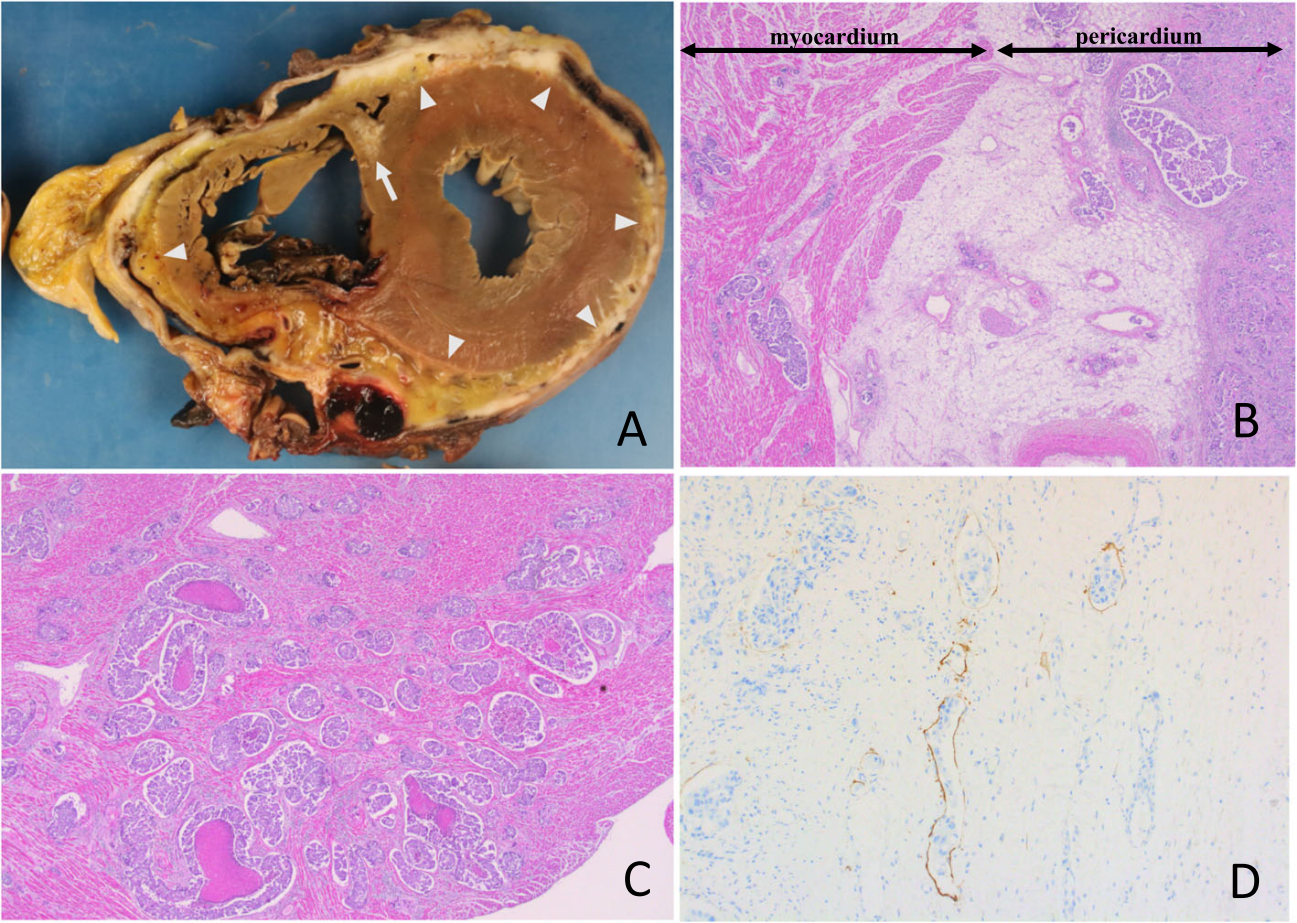
**a** Cancerous pericarditis: the parietal pericardium and visceral pericardium adhered to each other (*arrow heads*) and exhibited tumor infiltration (*arrow*). **b** Hematoxylin and eosin-stained pericardial tissue. A large number of tumor cells infiltrated the pericardium. Tumor cells infiltrated the lymph vessels of the myocardium (× 20). **c** Hematoxylin and eosin-stained pericardial tissue. A large number of tumor cells infiltrated the myocardium (× 20). **d** Immunostained (D2–40) pericardial tissue. Findings of pericardial lymphatic invasion and lymphatic metastasis (× 100)

In this case, a pathological autopsy was performed. On macroscopic examination, the parietal pericardium and visceral pericardium had adhered, indicating cancerous pericarditis (Fig. 2a). Pericardial effusion was not observed. On microscopic examination, a large number of tumor cells had infiltrated the pericardium, and some had infiltrated the lymph vessels of the myocardium (Fig. 2b and c). These results suggest pericardial metastasis of pancreatic cancer.

## Discussion

Carcinomatous pericarditis is observed in 10–20% of patients with malignancies, with the most common malignancies being lung cancer, breast cancer, leukemia, and malignant lymphoma. According to a study by Berge and Sievers, malignant tumor metastasis involving the pericardium was observed in 50% of autopsies of patients with metastatic malignant tumors [3]. Pericardial metastasis does not frequently cause cardiac tamponade. However, cardiac tamponade is a life-threatening condition requiring prompt diagnosis and treatment. Cardiac tamponade is the lethal phase of neoplastic pericarditis resulting from cardiac compression and the accumulation of pericardial fluid. Intrapericardial pressure increases and cardiac relaxation is inhibited, leading to low output; patients often experience dyspnea, hypotension, tachycardia, cold sweats, and fatigue [4]. When symptoms occur, echocardiography should be performed since it is the most useful examination for diagnosing pericardial effusion and detecting signs of tamponade. Although the diagnosis of cardiac tamponade is based on established clinical criteria, an accurate and early diagnosis of tamponade can often be established using echocardiography [5].

When neoplastic cardiac tamponade is diagnosed, drainage by pericardiocentesis should be performed to relieve symptoms and prevent sudden death. A high rate of recurrence (40–70%) has been reported due to the persistence of carcinomatous pericarditis-induced pericardial effusion [6]. Therefore, systemic and localized treatment should be provided to suppress pericardial effusion. Localized treatment includes percutaneous drainage, anticancer agent injection to destroy pericardial adhesions, and surgical resection of the pericardium. The most appropriate procedure should be selected based on the patient’s general condition. Intrapericardial treatment should be tailored to the type of tumor. For example, tris(1-aziridinyl)phosphine sulfide (Thiotepa) is recommended for patients with breast cancer, and cisplatin is recommended for patients with lung cancer [7]. The course after pericardial drainage has been reported to vary according to the type of cancer [4].

Malignant pericardial effusion is associated with an unfavorable prognosis. Wang *et al.* and Gross *et al*. reported that the mean survival times after detection of malignant pericardial effusion by pericardiocentesis were 3.1 and 3.7 months, respectively [8, 9]. To the best of our knowledge, no reports of intrapericardial treatment or endocardial treatment in patients with pancreatic cancer are available. We continued only systemic treatment because intrapericardial treatment could have caused diastolic dysfunction.

Several mechanisms regarding primary tumor metastasis to the pericardial cavity have been proposed, including lymphatic spread, hematogenous spread, systemic spread via nerves, and direct extension. Most tumors metastasize to the heart through the mediastinal lymph nodes. An autopsy was performed in this case, and a main lesion in the head of the pancreas with extensive fibrosis was observed. Moreover, active tumor cells remained. Vessel invasion and nerve invasion were observed, which were considered to have caused the metastasis to other organs. In the heart, the pericardium and myocardium showed strong tumor adhesion, which was considered indicative of cancerous pericarditis. Tumor cell infiltration was observed around almost the entire pericardium, and extensive lymphatic and nerve invasion was observed. Tumor cells also spread to the lymphatic vessels in the heart parenchyma, and some had infiltrated between myocardial fibers. Reports indicate that aminoglycoside antibacterial agents reduced cardiac contractility [10]. In this case, these types of antibiotics were not used, and the occurrence of cardiac dysfunction was considered unlikely. From the pathological results, the infiltration of cancer cells into the myocardium was considered remarkable, and the progression of the cancer caused a decrease in cardiac function, ultimately leading to death. Similar to that in the pericardium, severe lymphatic invasion and venous invasion were observed in several organs. Therefore, the tumor was considered to have reached the pericardium via a lymphatic or hematogenous route where it formed an infiltrating lesion. In the lung, metastasis formation, intravascular tumor embolism, and pleural dissemination were observed. The bronchiolar and bronchial walls showed high degrees of tumor invasion, and the peripheral airway was constricted, likely causing respiratory failure. Debilitation occurred throughout the body due to cachexia. Hepatic dysfunction occurred due to tumor infiltration into the liver. Thus, in this case, the cause of death was considered tumor metastasis, including pericardial metastasis, throughout the body. Pericardial metastasis of pancreatic cancer may suggest cancer metastasis throughout the whole body.

## Conclusion

Pericardial metastasis and cardiac tamponade in pancreatic cancer are extremely rare. In this case, lymphatic and vascular invasion caused metastasis to the pericardium and myocardium. In our patient, the interval between tamponade and death was very short (42 days). Pericardial metastasis may be an indication of systemic metastases, and tamponade can be considered an indicator of a poor prognosis. These possibilities are important to consider when pericardial effusion develops in patients with pancreatic cancer.
